# The effects of nutrients on stream invertebrates: a regional estimation by generalized propensity score

**DOI:** 10.1186/s13717-018-0132-x

**Published:** 2018-06-04

**Authors:** Zutao Ouyang, Song S. Qian, Richard Becker, Jiquan Chen

**Affiliations:** 1 Center for Global Change and Earth Observations, Michigan State University, 1405 S. Harrison Road, East Lansing, MI 48823 USA; 2 Department of Environmental Sciences, University of Toledo, 2801 West Bancroft Street, Toledo, OH 43606 USA

**Keywords:** Nutrient criteria, Water quality, Environmental management, Ecoregion

## Abstract

**Introduction:**

The effects of nutrients on stream conditions within individual streams or small areas have been studied extensively, but the same effects over a large region have rarely been examined due to the difficulty of applying large-scale manipulative experiments. In this study, we estimated the causal effects of nutrients within the Western United States on invertebrate richness, an important biological indicator of stream conditions, by using observational data.

**Methods:**

We used the generalized propensity score method to avoid the common problem of statistical inference using observational data, i.e., correlation established based on observational data does not imply a causal relationship because the effects of confounding factors are not properly separated.

**Results:**

Our analysis showed a subsidy-stress relationship between nutrients and invertebrate taxon richness in the whole Western United States and in its sub-ecoregions. The magnitude of the relationship varies among these sub-ecoregions, suggesting a varying nitrogen effect on macroinvertebrates due, in large part, to the varying natural and anthropogenic conditions from ecoregion to ecoregion. Furthermore, our analysis confirmed that causal estimation results using regression can be sensitive to the imbalance of confounding factors.

**Conclusions:**

Stratifying data into ecoregions with relatively homogeneous environmental conditions or adjusting data by generalized propensity score can improve the balance of confounding factors, thereby allowing more reliable causal inference of nutrient effects. Invertebrates respond to the same nutrient levels differently across different site conditions.

## Introduction

Nutrients are essential for maintaining an ecosystem’s structure and function. Knowledge of the effects of excessive nutrients on ecosystems is important for environmental management. In streams, increased nutrient concentrations have altered biological structures and functions such as species richness, composition, abundance, and decomposition rate (Dodson et al. 2000; Freeman et al. 2009; Smith et al. 1999; Gulis and Suberkropp 2004; Rosemond et al. 1993). Excessive nutrients can also reduce water quality causing problems for drinking water and can deplete dissolved oxygen, leading to fish kills (USEPA 1996). For example, 40% of rivers in the USA have been impaired primarily as a result of excessive nutrients (USEPA 1996).

Invertebrates occupy an important ecological niche in streams, and their aggregated measures such as total taxon richness are widely used for stream condition assessments (Fore et al. 1996; Moss et al. 1987). However, while a few key invertebrate taxon grazers have been examined in many field and laboratory studies, relatively little work has been done to examine the effects of nutrients on aggregate measures of invertebrate assemblages (e.g., richness) (Yuan 2010; Cross et al. 2006; Quinn et al. 1997). As a result, our current understanding of the causal effects between increased nutrients and invertebrate richness (IR) is still limited.

How responsively does the IR change with nutrients, and does the causal relationship vary regionally? The causal relationship details are necessary to inform management actions and provide proper measures. A causal relationship is ideally quantified using manipulative experiments where treatments are randomly applied to replicated samples. This approach eliminates the effects of confounding factors by adequately resolving the counterfactual problem (Maldonado and Greenland 2002) in a causal analysis. Such manipulative studies (e.g., Cross et al. 2006; Gafner and Robinson 2007; Hart and Robinson 1990; Slavik et al. 2004) have increased our understanding of the effects of nutrients on streams but were usually conducted in a very small area or single whole stream/channel representing limited conditions. As a result, it is difficult to draw general conclusions from those studies for a region (e.g., the regional average effect) for setting regional nutrient criteria. Applying randomized experiments is difficult in the case of many streams across a large landscape. An alternative approach to study regional average effect is to use observational data that have been collected from many streams spanning different conditions/locations, which might produce complemental knowledge to that what we have gained from manipulative studies.

Observational data, by definition, are collected without a random sampling mechanism with respect to the effect of the variable of interest. When observational data are used without properly addressing potential problems induced by the non-random nature of the data collection process (e.g., imbalance of factors other than the variable of interest, i.e., confounding factors), results can be biased (Qian and Harmel 2016). However, rare studies in the ecological literature have addressed this problem of confounding factors, which can lead to divergent results of the same problem. For example, Clenaghan et al. (1998) reported a positive association between nutrients and benthic macroinvertebrates in one catchment in Ireland; Heino et al. (2003) reported the same positive association in a river in Finland; Bergfur et al. (2007) found a negative correlation in streams of central Sweden; Wang et al. (2007) demonstrated a negative association in some wadeable streams in Wisconsin; and Harding et al. (1999) and Niyogi et al. (2007) showed no clear link between macroinvertebrates and nutrient concentrations in one river and in a suite of 21 streams of southern New Zealand, respectively. These divergent results are expected, as these studies focused on local streams and each stream may have different confounding factors (e.g., watershed land use patterns, habitat quality, and flow conditions). Because nutrient criteria are usually developed for a large geographic region and not for individual streams or at a local level, understanding the regional (average) effects of nutrient enrichment is necessary. In this study, we aim to evaluate the regional average effects of nutrients on stream invertebrate taxon richness in the Western United States and its individual ecoregions from observational data.

An effect, or more specifically a causal effect, of a nutrient on invertebrate richness cannot be equated to the correlation between the two variables when the data used are observational data. In observational data, a treatment (i.e., nitrogen concentrations) is “assigned” to each site through some unknown and likely non-random processes. The resulting data are not balanced with respect to confounding factors. In other words, observed values of a confounding factor cannot be the same for streams with different observed treatment levels (nitrogen concentration). This imbalance often leads to a biased estimate of the causal relationship. One statistical approach to this problem is the use of propensity score matching (Rosenbaum and Rubin 1983; Rubin 2006). The propensity score matching approach was designed for binary treatment variables. It has been used for assessing the effectiveness of agricultural conservation practices on nutrient loss (Qian and Harmel 2016). In our study, the treatment variable, nitrogen concentration, is a continuous variable. We use the generalized propensity score method (Hirano and Imbens 2004; Imai and van Dyk 2004), which estimates the causal relationship by averaging out the effects of known confounding factors. The generalized propensity score for continuous treatments is an extension of the well-established and widely used propensity score methodology for binary treatments (Rosenbaum and Rubin 1983) and multivalued treatments (Rubin 2006).

Here, we used the generalized propensity score method of Hirano and Imbens (2004). This method does not presume any specific linear or nonlinear relationship and allows for flexibility. Our specific questions were “How does the invertebrate taxon richness change with increased eutrophication in the Western United States, and does this causal relationship vary by ecoregion?” Answers to these questions can provide baseline scientific information for nutrient criteria development.

## Methods

### Data

**Fig. 1:**
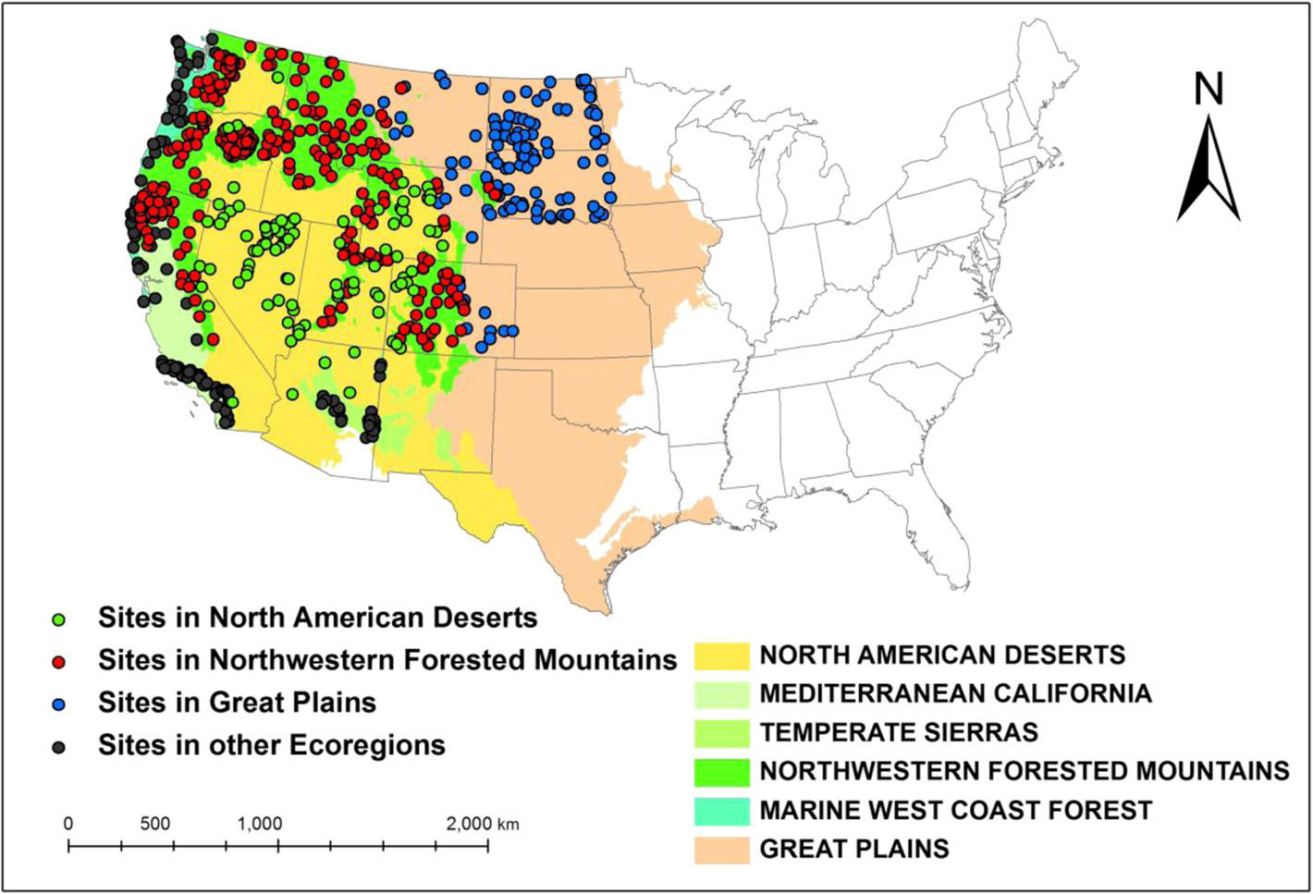
The sampled stream sites as well as their associated level I ecoregions defined by USEPA. Three subsets (strata) were extracted for analysis: the Great Plains’ streams (blue points), the Northwestern Forested Mountains’ streams (red points), and the North American Deserts’ streams (green points)

We used observational data collected by the USEPA at wadeable stream reaches from 12 western US states (Washington, Montana, North Dakota, South Dakota, Wyoming, Idaho, Oregon, Nevada, Utah, California, Colorado, and Arizona) during the summers of 2000–2002 (Fig. 1) (Stoddard et al. 2006). Randomness was achieved through a probability-based sample design under the Environmental Monitoring and Assessment Program (EMAP) (Blair 2001). Extensive biological, physical, chemical, and landscape-scale measurements were collected at each sampled site (USEPA 2000), but we only used those related to our study (Table 1). In total, 670 randomly sampled stream sites that had a complete observation of these variables were included in this study. We used total invertebrate taxon richness as our response variable. Invertebrate richness (IR) was measured as the total number of distinct invertebrate taxa observed in each sample.

**Table 1:** Covariates of total nitrogen (TN) included in the study and their correlation with log (TN) within all streams

Variable	Description	Units	*r*
ELEV	Elevation	m	− 0.18
Longitude	Longitude	Degree	0.57
Log(PRECIP)	Annual precipitation in log scale	mm	− 0.58
Log(AREA)	Catchment area in log scale	km^2^	0.47
Log(CL)	Cl^−^ concentration in log scale	μg/L	0.62
Log(HCO3)	HCO_3_^−^ concentration in log scale	μg/L	0.55
Log(SO4)	SO_4_^−2^ concentration in log scale	μg/L	0.61
SED	Sand and fine substrate in the stream (< 2 mm in diameter)	μg/L	0.63
STRMTEMP	Stream temperature	Degree	0.49
Percent.AGT	Percentage of catchment in agricultural land use	μg/L	0.57
Percent.URB	Percentage of catchment in urban land	Percentage	0.30
Percent.Canopy	Percentage of open canopy	Percentage	0.47
Riparian.Disturb	Riparian agricultural disturbance index	Index	0.49

All correlation coefficients are statistically significant (*p* < 0.05)

**Fig. 2:**
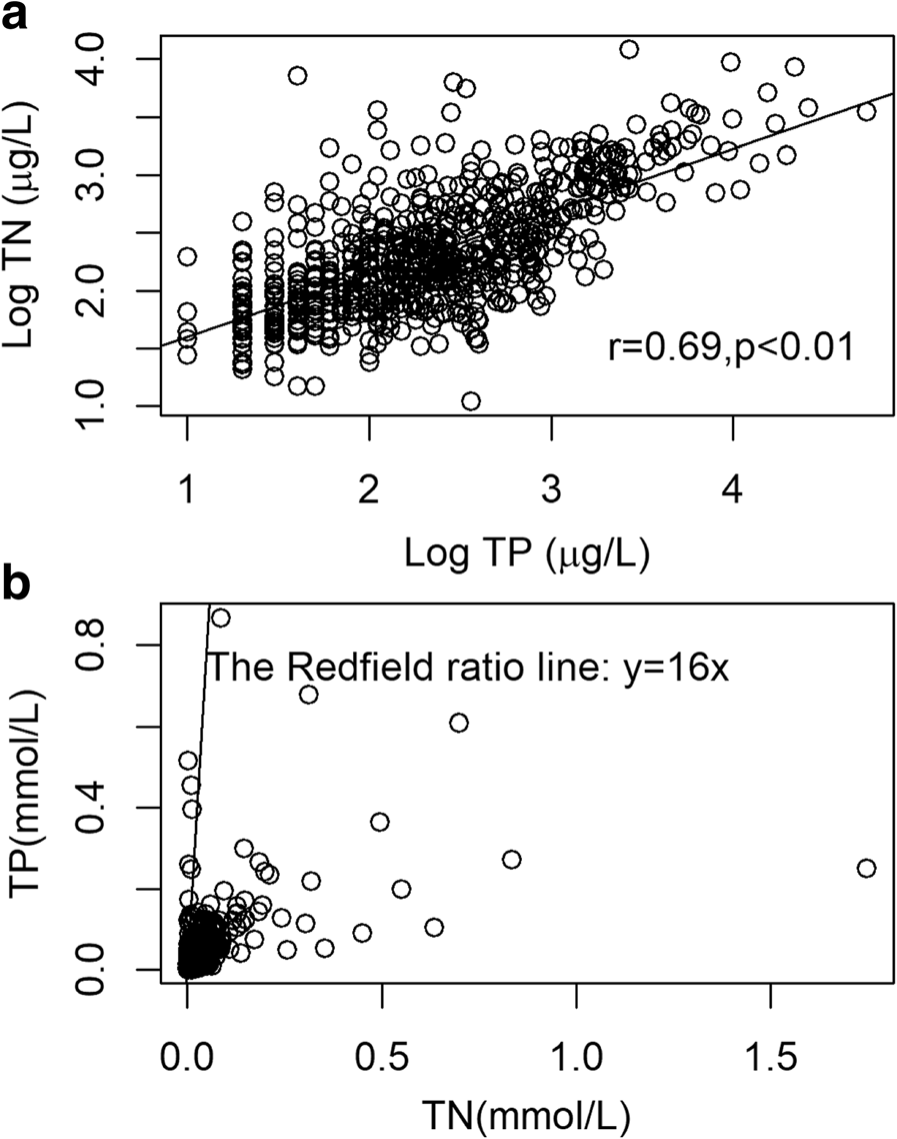
The correlation between total nitrogen and total phosphorus at log scale (**a**) and the stoichiometric relationship (**b**)

Nutrient conditions in these streams are represented by total nitrogen (TN, in μg/L). It is used as the treatment variable. Stream periphyton could either be nitrogen (N) or phosphorus (P) limited, but both P and N additions stimulate periphyton growth (Francoeur 2001; Elser et al. 2007). Total P is highly correlated with TN in this dataset (Fig. 2a), and the stoichiometric ratio of N:P in our dataset is mostly below the Redfield ratio (Fig. 2b), suggesting N limitation. Therefore, we assumed that both TP and TN influenced stream biota and that TN concentrations can represent the effects of both nutrients across this wide range of streams in our study.

We considered 13 important covariates (variables that co-varied with nutrient concentration) as major confounding factors of the TN-IR dose-response relationship (Table 1). Covariates were identified by examining bivariate scatter plots and by investigating the correlation between TN and each candidate variable. We used variables with statistically significant correlation with TN (*p* < 0.05). Methods of field collection and variable extraction of the 13 covariates can be found in Yuan (2010) and Stoddard et al. (2006). TN, precipitation, and other chemistry measurements (e.g., chloride ion) were highly skewed and were thus log-transformed (base 10) prior to analysis (Table 1).

### Data stratification

When using data from regions where the average natural conditions are systematically different, different TN-IR dose-response models may be produced. We illustrated this by stratifying the data into level I ecoregions of North America (Omernik 1987) (Fig. 1). Our full dataset fell into six different ecoregion categories. However, we only included the three ecoregions (strata) that had a sample size large enough to run our models. Stratum one included streams in the Northwestern Forested Mountains (*n* = 345), stratum two included all streams within the Great Plains (*n* = 120), and stratum three included all streams within the North American Deserts (*n* = 99). These three ecoregions have different natural conditions as well as different levels/types of human activities (Table 2) and are thus good candidates to demonstrate regional divergence.

**Table 2:** The mean and standard deviation of all confounding factors, the treatment log TN, and the response invertebrate richness within all streams and within the three strata

Variable	Means ± standard deviation			
	All streams	Forested Mountains	Great Plains	North American Deserts
Log(TN)	2.33 ± 0.51	2.04 ± 0.35	2.95 ± 0.36	2.64 ± 0.38
Invertebrate richness	48.30 ± 15.15	55.19 ± 12.58	33.72 ± 11.76	40.03 ± 11.49
ELEV	1252.06 ± 722.10	1455.84 ± 688.03	783.80 ± 379.71	1701.97 ± 501.16
Longitude	− 114.04 ± 7.55	− 166.85 ± 5.48	− 102.06 ± 3.16	− 113.58 ± 4.06
Log(PRECIP)	− 0.31 ± 0.56	− 0.07 ± 0.49	− 0.81 ± 0.18	− 0.74 ± 0.27
Log(AREA)	4.07 ± 2.34	3.09 ± 1.68	6.77 ± 1.73	4.53 ± 2.56
Log(CL)	4.35 ± 1.85	3.05 ± 1.20	6.04 ± 1.21	5.28 ± 1.73
Log(HCO3)	7.34 ± 1.12	6.76 ± 1.06	8.44 ± 0.55	7.75 ± 0.77
Log(SO4)	5.48 ± 2.42	3.97 ± 1.52	8.70 ± 1.61	6.42 ± 1.90
SED	35.45 ± 30.35	1.82 ± 8.95	12.79 ± 32.54	4.85 ± 20.45
STRMTEMP	15.16 ± 5.55	12.39 ± 4.19	20.58 ± 5.02	16.08 ± 5.70
Percent.AGT	8.10 ± 21.28	0.58 ± 5.59	38.72 ± 33.44	2.90 ± 9.58
Percent.URB	0.17 ± 0.47	0.06 ± 0.22	0.33 ± 0.44	0.13 ± 0.47
Percent.Canopy	0.37 ± 0.38	0.23 ± 0.31	0.72 ± 0.32	0.59 ± 0.39
Riparian.Disturb	0.56 ± 0.64	0.36 ± 0.57	1.10 ± 0.53	1.03 ± 0.56

### An overview of statistical methods for causal inference

The fundamental concept in causal inference is the concept of counterfactual, which requires that the responses to treatment and control be measured from the same subject (Maldonado and Greenland 2002). For example, assessing the causal effect of TN ideally requires the quantification of the IR increase/decrease due to the only change in TN, which means that we have to compare potential outcome IR observed at two different levels of TN (e.g., 0.1 mg/L, 0.2 mg/L) under identical conditions (e.g., the same site and the same moment) to avoid any confounding factors. However, only one of the two potential outcomes can be actually observed; therefore, it is the counterfactual. The statistical solution to the counterfactual problem is Fisher’s randomized experiments (Fisher 1966), such that the average effect of a treatment can be quantified with a reasonable level of confidence. The propensity score matching method of Rosenbaum and Rubin (1983) is the most widely used method for causal inference with a binary treatment for observational data. The propensity score is defined as the conditional probability of a subject receiving the treatment given all observed covariates and is modeled as a function of the observed covariates. Instead of matching observed units either directly or by using a nearest-neighbor method in multiple dimensions, this propensity score makes matching possible in one dimension. Rosenbaum and Rubin (1983) proved that matching and sub-classifying with propensity scores can sufficiently remove bias due to confounding factors (see Qian and Harmel (2016) for an example of environmental application). Hirano and Imbens (2004) and Imai and van Dyk (2004) extended the propensity score method to include a continuous treatment variable, which they called a generalized propensity score (GPS). Like the propensity score, a GPS is defined as the conditional probability density function of the treatment given the covariates:

1 $$ r\left(t,x\right)={f}_{\left.T\right|X}\left(\left.t\right|x\right) $$

where *f* is the probability density function and *T* is the treatment set, *X* is the covariate(s), and *t*∈*T* and *x*∈*X*. Under a weak unconfoundedness assumption (which implies that all important confounding factors are included in the model for deriving the propensity score), Hirano and Imbens (2004) showed that the GPS is a balancing score, which means that sample units with similar propensity scores have similar covariates independent of treatment levels. Hence, grouping sampling units with similar generalized propensity scores is an effective means of removing or reducing confounding effects (Hirano and Imbens 2004; Imai and van Dyk 2004).

### Implementation of the generalized propensity score

We implemented the generalized propensity score method presented in Hirano and Imbens (2004) to both the full dataset and the three strata. First, we used regression to derive the probability distribution of the treatment variable (log(TN)). This is a purely mathematical step. The distribution (*f*(*t*|*x*)) in Eq. (1) is usually developed using an empirical (e.g., linear regression) model:

2 $$ T= X\beta +\varepsilon $$

where *T* represents the treatment (log(TN)), *X* represents the vector of covariates (Table 1), and *ε* is the residual term assumed to follow a normal distribution N (0, *σ*). The regression coefficients and *σ* are estimated by using maximum likelihood. After fitting the model, the model’s residuals *σ* were used as a measure of how well each observed log(TN) was predicted by the covariates. Probability densities of these residuals are:

3 $$ r\left({T}_i,{X}_i\right)=\frac{1}{\sqrt{2\pi {\widehat{\sigma}}^2}}\exp \left(-\frac{1}{2{\widehat{\sigma}}^2}{\left({T}_i-X\widehat{\beta}\right)}^2\right) $$

The generalized propensity score for each observation (*i*) is the probability density *R*_*i*_ = *r*(*T*_*i*_, *X*_*i*_) (i.e., the likelihood of receiving treatment *T*_*i*_, given observed covariates *X*_*i*_), which was used to account for the aggregated effects of all the covariates in Eqs. (4) and (5). We emphasize here that the generalized propensity score is not the predicted treatment outcome, as in Eq. (2), but instead was the probability density of the residuals of the prediction, as in Eq. (3).

The second step is the estimation of the expectation of the outcome IR conditional on the observed covariates and treatment levels. For each observation, we have the calculated generalized propensity score *R* that represents the effects of all confounding factors and expect that the response variable is a function of both the treatment and confounding factors; therefore, we can use a polynomial regression model to approximate the functional dependency of IR on *T* and *R*:

4 $$ E\left[\left.{Y}_i\right|{T}_i,{R}_i\right]={a}_0+{a}_1\cdot {T}_i+{a}_2\cdot {T}_i^2+{a}_3\cdot {R}_i+{a}_4\cdot {R}_i^2+{a}_5\cdot {T}_i\cdot {R}_i $$

where *E* is the expectation operator. The parameters in Eq. (4) are estimated by ordinary least squares. This process is similar to conventional multiple regression where the covariates are included as predictors. As a result, it is still too early to interpret the regression in Eq. (4) as a causal effect (Hirano and Imbens 2004), because the conditional expectation of the outcomes is still conditional on the observed covariates (e.g., represented by GPS) which are different among all units, and a causal interpretation should compare expected outcomes with the same score but different treatment levels. One approach is to divide samples into groups with similar scores and then apply Eq. (4) to approach causal interpretation by averaging the coefficients from the groups (Imai and van Dyk 2004). Alternatively, Hirano and Imbens (2004) suggested that the average dose-response model could be estimated by integrating out the aggregated confounding factor represented by *R*_*i*_. Numerically, we can approximate the integration by averaging equation over *R*_*i*_ evaluated at a series of treatment levels. That is, for a given log(TN) concentration *t*, the average treatment effect is:

5 $$ u(t)=\widehat{E}\left[Y(t)\right]=\frac{1}{N}\sum \limits_{i=1}^N\left({\widehat{a}}_0+{\widehat{a}}_1\cdot t+{\widehat{a}}_2\cdot {t}^2+{\widehat{a}}_3\cdot \widehat{r}\left(t,{X}_i\right)+{\widehat{a}}_4\cdot \widehat{r}{\left(t,{X}_i\right)}^2+{\widehat{a}}_5\cdot \widehat{r}\left(t,{X}_i\right)\cdot t\right) $$

where *u*(*t*) is the average potential outcome at treatment level *t*, $\widehat{r}\left(t,{X}_i\right)$ is calculated based on Eq. (3) (substituting *T*_*i*_ with *t*), and *N* is the sample size of the data, and the parameters ${\widehat{a}}_0$, ${\widehat{a}}_0$, ${\widehat{a}}_0$, ${\widehat{a}}_0$, and ${\widehat{a}}_0$ are estimated from the second step using Eq. (4).

### Verification of generalized propensity score

The generalized propensity score is a balancing score (Hirano and Imbens 2004; Imai and van Dyk 2004) when the model specification is appropriate. In other words, when observations are grouped into subsets with similar propensity scores, covariates within a subset should be similar among different treatment levels when the model used to derive the scores is appropriate. We use this expectation to verify the adequacy of the model specification. Hirano and Imbens (2004) proposed the use of a standardized difference to measure the balance in two steps. First, data were grouped into a number of categories (e.g., three) based on log TN (the treatment), each with roughly the same sample size. To show the balance in a confounding factor, we compare the means of the confounding factor among the three categories. The comparison can be made using a standardized difference, the difference of the means in the two categories divided by the standard error of the difference. The standard difference is similar to the *t* statistics in a two-sample *t* test. This comparison is first carried out directly to the data without GPS adjustment to illustrate the imbalance in the data. After calculating the propensity scores, we then divide the data into subsets of similar propensity scores and repeat the process of comparing confounding factors among three log TN categories within each relatively homogeneous subset of data. That is, in the second step, the data were divided into a number of subsets based on the calculated propensity scores (confounding factors are balanced within each subset), and within each subset, we further divide the data based on the treatment (log TN) to calculate the standardized mean differences of each confounding factor. The weighted averages of these standardized mean differences are known as the GPS-adjusted mean differences (Hirano and Imbens 2004). Weights of each subset are determined by the subset sample sizes. When the standardized differences of a confounding factor are between − 2 and 2, we consider that the confounding factor is balanced. Through comparing the change in the standardized differences calculated from the two steps, we can show the improvement in terms of confounding factor balance due to propensity score.

### Comparison with regression without propensity score adjustment

We compared the dose-response estimation from the generalized propensity score to conventional regression models (without using propensity score adjustment). For the entire dataset, the Forested Mountains stratum and the North American Deserts stratum, we fitted generalized additive models (GAM) to compare with the generalized propensity score because of the nonlinear subsidy relationship. The selection of a smoothness parameter impacts the result of GAM. We used the default smoothness parameter in R package mgcv, which is determined based on a cross-validation simulation for optimal predictive features. The relationship was approximately linear for the Great Plains stratum. We thus fitted the simple linear and multiple linear regressions for comparison, which were then compared with the dose-response function estimated by generalized propensity score. 95% confidence zone of estimation from the generalized propensity scores was computed from a 1000 times bootstrap analysis. The statistical software used for all analyses was R version 3.24.

## Results

### Balancing check

The imbalance of confounding factors is shown by the standardized differences of the means of these variables among data groups with different log TN levels (Tables 3, 4, 5, and 6). The level of imbalance also varies by ecoregion. For example, data for the Great Plains stratum and North American Deserts are roughly balanced without generalized propensity score adjustments (Tables 5 and 6). Adjusting data by GPS score apparently increased the balance of confounding factors, especially for the dataset as a whole (Table 3).

**Table 3:** Standardized mean differences between one group and the other two combined, using the entire dataset

Variable	Grouped by log TN (unadjusted)			Adjusted by GPS		
	[1.17, 1.78]	[1.78, 2.69]	[2.69, 4.08]	[1.17, 1.78]	[1.78, 2.69]	[2.69, 4.08]
ELEV	− 1.33	6.88	6.68	0.32	2.40	1.57
Longitude	− 7.78	− 7.07	− 15.73	− 1.94	0.51	− 1.79
Log(PRECIP)	12.06	2.98	13.20	0.79	− 0.07	2.61
Log(AREA)	− 4.73	− 7.02	− 12.46	− 1.85	− 0.74	− 0.76
Log(CL)	− 9.14	− 5.40	− 14.43	− 1.88	1.47	− 2.41
Log(HCO3)	− 9.28	− 4.77	− 13.59	− 0.94	− 1.52	− 3.24
Log(SO4)	− 7.74	− 8.00	− 17.23	− 0.76	− 0.63	− 4.16
SED	− 6.67	− 8.77	− 17.24	− 1.76	− 0.36	− 2.33
STRMTEMP	− 6.67	− 5.15	− 11.72	−0.64	0.26	− 1.32
Percent.AGT	− 3.71	− 12.21	− 18.96	− 1.15	− 4.67	− 7.54
Percent.URB	− 2.86	− 2.91	− 5.58	0.89	0.61	− 0.55
Percent.Canopy	− 5.72	− 5.40	− 11.19	− 1.72	0.56	− 1.14
Riparian.Disturb	− 8.04	− 3.88	− 11.15	1.11	0.60	− 1.85

The “Grouped by log TN” columns compare confounding factors in the three subsets divided by three continuous intervals of log TN (in brackets), each with about 1/3 of the total samples. The “Adjusted by GPS” columns compare the confounding factors based on GPS in two steps: (1) data were divided based on GPS into six groups and (2) data within each GPS group were further divided into three groups based on the same log TN brackets, and mean differences were calculated. The sample size-weighted averages of these mean differences are shown

**Table 4:** Standardized mean differences between one group and the other two combined for the Forested Mountains ecoregion stratum

Variable	Grouped by log TN (unadjusted)			Adjusted for the GPS		
	[1.17, 1.60]	[1.60, 2.44]	[2.44, 3.74]	[1.17, 1.60]	[1.60, 2.44]	[2.44, 3.74]
ELEV	− 2.73	0.74	− 1.53	− 0.49	0.57	1.29
Longitude	− 3.43	0.36	− 2.65	− 0.51	0.43	1.15
Log(PRECIP)	5.12	− 0.16	4.41	0.63	− 0.93	− 0.28
Log(AREA)	− 1.56	0.24	− 1.12	0.44	− 0.72	0.89
Log(CL)	− 2.81	− 3.42	− 7.35	− 1.01	− 0.95	− 3.39
Log(HCO3)	− 3.62	− 0.69	− 4.22	− 0.64	− 1.09	− 0.29
Log(SO4)	− 3.26	− 0.13	− 3.13	− 1.61	− 0.44	− 0.36
SED	0.54	− 1.61	− 1.56	− 0.06	− 1.34	0.03
STRMTEMP	− 2.30	0.22	− 1.80	− 0.21	− 0.50	0.57
Percent.AGT	− 0.60	− 1.90	− 2.98	− 0.23	0.34	− 5.56
Percent.URB	− 0.74	− 2.21	− 3.53	0.26	− 0.16	− 6.47
Percent.Canopy	− 1.41	− 2.37	− 4.40	− 0.99	− 0.14	− 1.32
Riparian.Disturb	− 3.43	− 2.41	− 6.50	− 1.64	− 1.21	− 1.52

See Table 3 for explanations

**Table 5:** Standardized mean differences between one group and the other two combined for the Great Plains ecoregion

	Grouped by log TN (unadjusted)			Adjusted for the GPS		
Variable	[2.05, 2.56]	[2.56, 3.19]	[3.19, 4.08]	[2.05, 2.56]	[2.56, 3.19]	[3.19, 4.08]
ELEV	7.45	− 1.37	− 9.02	1.85	0.30	0.91
Longitude	− 5.37	− 0.05	− 3.92	− 1.65	0.98	− 2.14
Log(PRECIP)	3.15	− 2.94	− 0.87	2.08	− 2.17	− 1.39
Log(AREA)	− 3.46	2.02	− 0.29	− 1.63	1.89	1.22
Log(CL)	0.63	− 1.31	− 0.96	0.69	− 1.48	− 1.42
Log(HCO3)	− 4.67	1.04	− 2.13	− 1.55	0.19	− 0.98
Log(SO4)	− 2.22	0.11	− 1.53	0.45	− 0.37	− 0.46
SED	0.07	− 2.21	− 2.41	2.38	− 1.41	− 2.08
STRMTEMP	− 3.59	0.99	− 1.49	− 1.50	0.26	− 0.81
Percent.AGT	− 4.05	− 0.78	− 3.98	− 0.52	− 1.13	− 1.87
Percent.URB	1.05	− 1.62	− 0.99	0.21	− 1.80	− 1.74
Percent.Canopy	0.16	0.20	0.35	− 0.14	0.39	1.05
Riparian.Disturb	− 2.36	2.19	0.65	− 1.52	1.83	0.98

See Table 3 for explanations

**Table 6:** Standardized mean differences between one group and the other two combined for the North American ecoregion

Variable	Unadjusted			Adjusted for the GPS		
	[1.77, 2.35]	[2.35, 2.69]	[2.69, 3.80]	[1.77, 2.35]	[2.35, 2.69]	[2.69, 3.80]
ELEV	1.24	− 1.95	− 0.71	0.74	− 2.57	0.14
Longitude	0.35	− 0.64	− 0.30	0.77	− 0.57	− 0.95
Log(PRECIP)	2.82	0.27	3.10	0.79	0.52	2.28
Log(AREA)	− 1.23	2.17	0.93	− 0.62	1.99	1.95
Log(CL)	− 3.75	1.30	− 2.23	− 2.35	1.53	− 1.89
Log(HCO3)	− 0.65	− 0.71	− 1.37	1.08	− 1.05	− 0.89
Log(SO4)	− 1.66	0.87	− 0.75	0.59	1.31	− 1.17
SED	− 1.21	− 0.67	− 1.90	− 1.49	− 0.23	− 1.66
STRMTEMP	− 2.69	2.35	− 0.27	− 2.03	1.71	1.38
Percent.AGT	− 0.89	− 1.35	− 2.29	− 0.43	− 1.59	− 3.11
Percent.URB	− 0.94	1.11	0.19	− 0.61	2.55	− 0.73
Percent.Canopy	− 1.11	− 0.31	− 1.43	− 0.72	− 0.66	− 1.05
Riparian.Disturb	− 1.02	0.22	− 0.79	0.72	0.57	0.56

See Table 3 caption for explanation

### Dose-response relationship

**Fig. 3:**
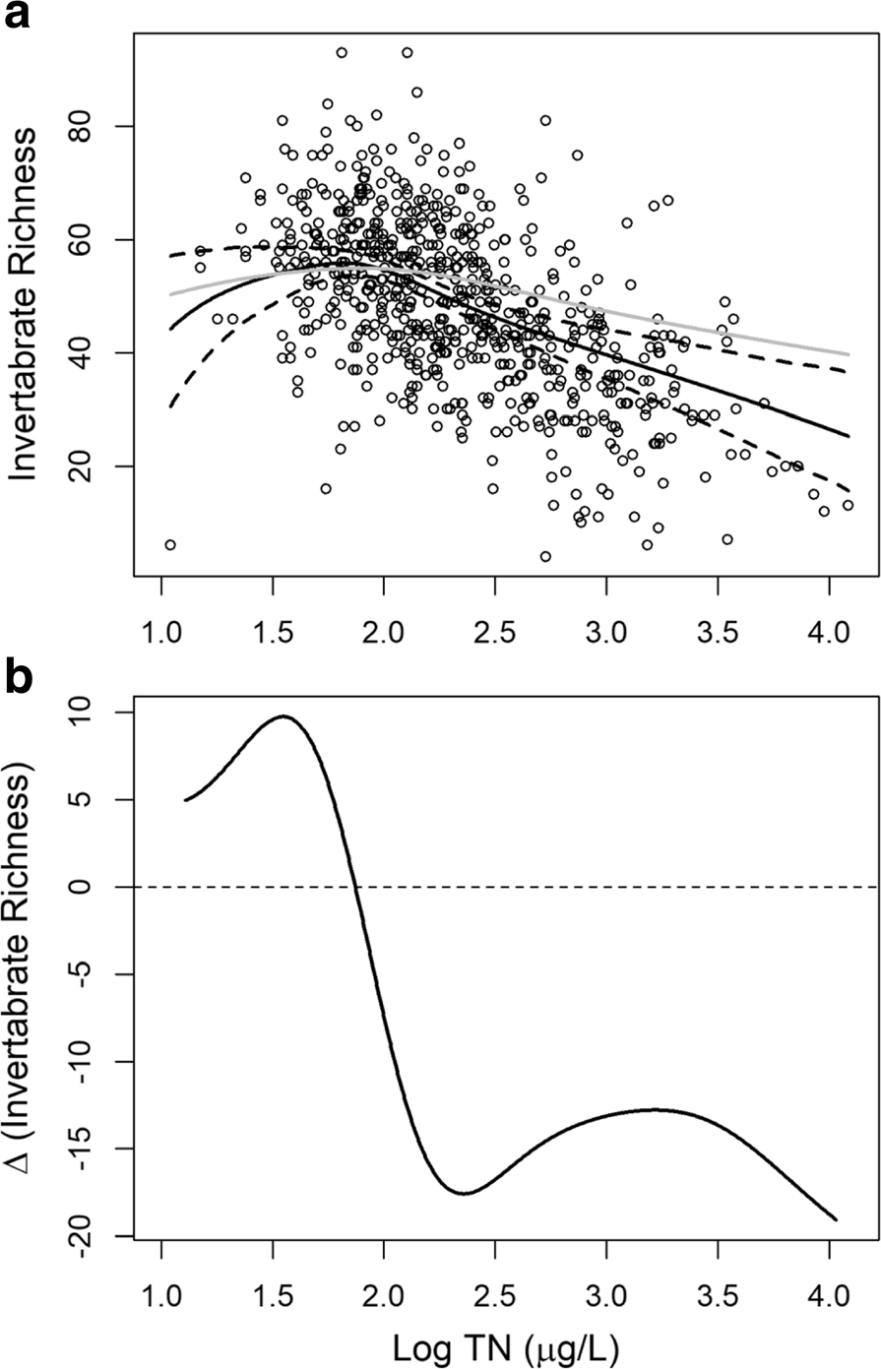
**a** The regional causal effects of total nitrogen on invertebrate richness in the Western United States. The black solid line and the black dashed lines are the mean estimations by generalized propensity score of the TN-IR dose-response function and the corresponding 95% confidence boundaries, respectively. The gray solid line is the estimation by GAM. **b** The change rate (slope) of invertebrate richness at different nitrogen levels

A subsidy-stress relationship was observed between log(TN) and IR in the Western United States (Fig. 3a). The total N concentrations in streams of the 12 states covered a wide range of concentrations (log(TN) from 1.04 to 4.19, or TN from 11 to 15,625 μg/L). Across this wide geographical region, nutrients first affected invertebrate richness positively and then gradually switched to a negative effect, with a breaking point log TN at ca. 1.80 (63.09 μg/L) (Fig. 3b).

**Fig. 4:**
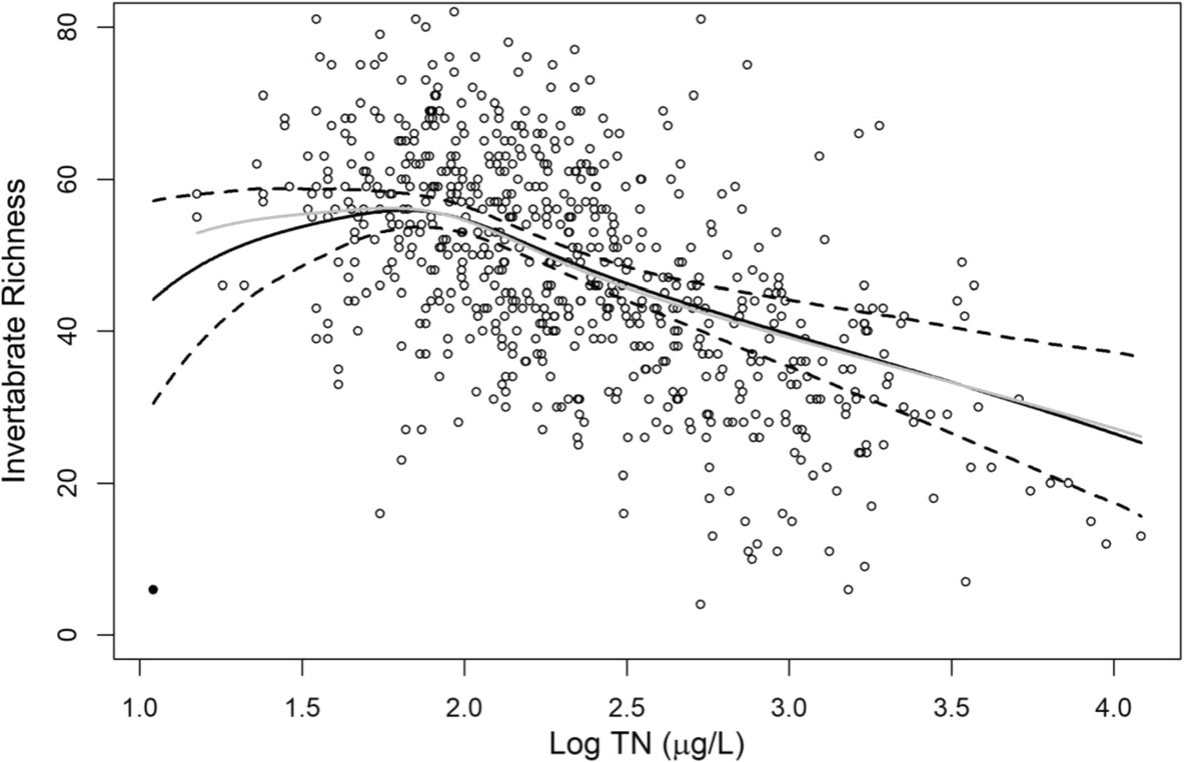
Comparison between the estimated effects of total nitrogen on invertebrate richness based on the generalized propensity score with/without the “outlier” stream site (the black point). The black solid line and the black dashed lines are the mean estimations and the corresponding 95% confidence boundaries including the outlier, respectively. The gray line is the mean estimation excluding the outlier

One stream site with a low total N concentration and the lowest invertebrate richness (Fig. 3a) seemed to be a leveraging data point at the low end of the nitrogen gradient. When the data point is removed, the result did not change significantly (Fig. 4). Additionally, this subsidy-stress relationship was not a result of the polynomial model (Eq. (4)). Using a first order polynomial instead of the second order polynomial as in Eq. (4) resulted in a similar dose-response relationship, shown in Fig. 3a.

**Fig. 5:**
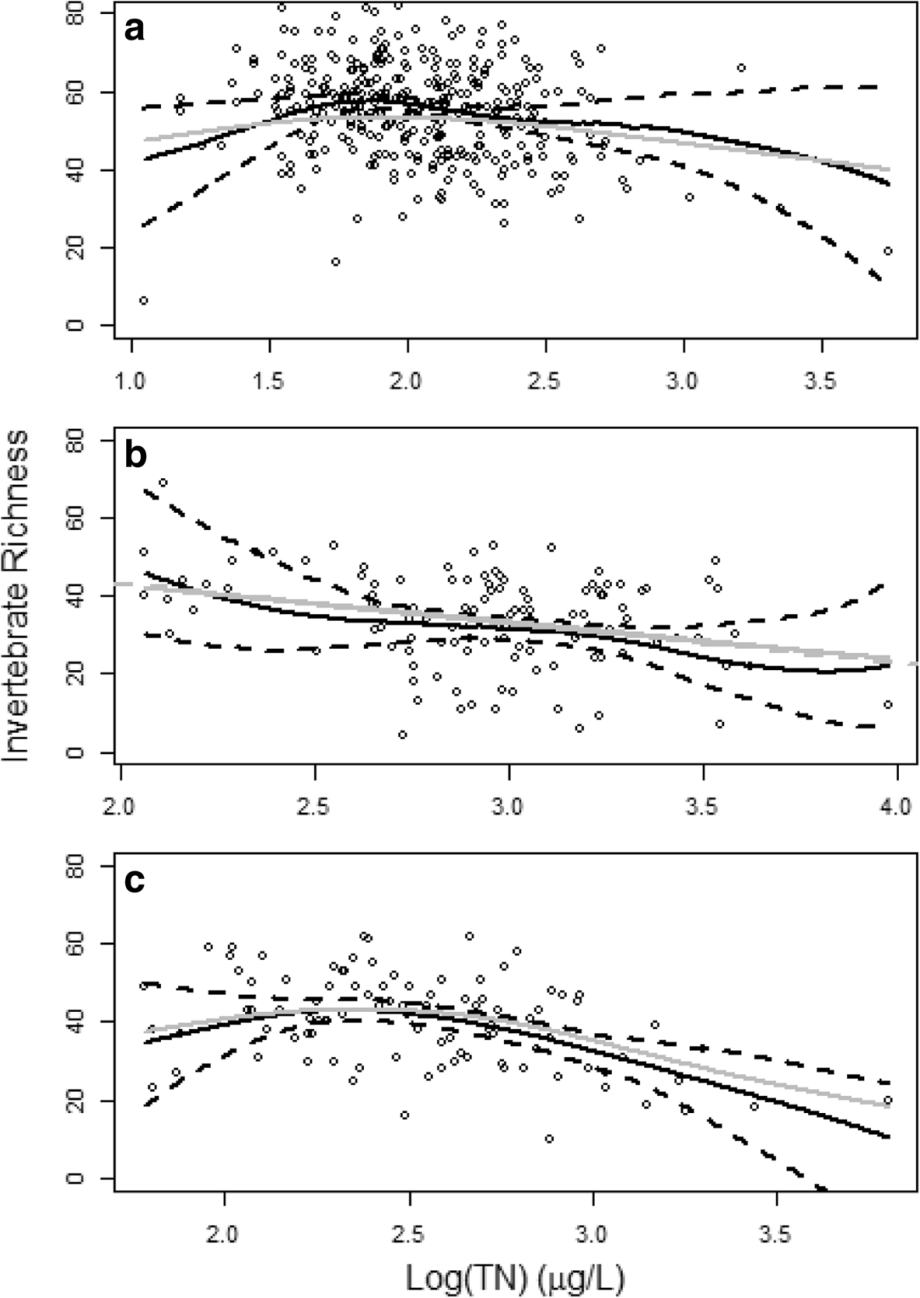
The causal effects of total nitrogen on invertebrates in different ecoregions of the Western United States. **a** Forested Mountains. **b** Great Plains. **c** North American Deserts. The black solid lines and black dashed lines are the mean estimations by generalized propensity score of the TN-IR dose-response function and the corresponding 95% confidence boundaries, respectively. The gray solid lines are the estimations by the general additive model in **a** and **c** and the estimations by simple linear regression in **b**. The gray dashed line in **b** is the estimation by multiple linear regressions and nearly overlaps the simple linear regression line

For streams in the Northwestern Forested Mountains, a subsidy-stress relationship was observed with an optimal log(TN) similar to the same tipping point from the model for the entire study area (Fig. 5a) but with higher IR at the same nutrient level. For streams in the Great Plains, nutrient levels were high in all streams due to heavy agriculture. As a result, a monotonously negative relationship was observed between nitrogen and invertebrate richness (Fig. 5b). A subsidy-stress relationship was also observed in North American Deserts but with a different optimal N concentration (Fig. 5c). The same negative relationship was present among different ecoregions when nutrient levels were high, suggesting that nitrogen is a stressor to invertebrates when at high concentrations. Among the three ecoregions, streams in the Great Plains had the lowest invertebrate richness when at the same nutrient level; the streams in the Northwestern Forested Mountains had the highest invertebrate richness.

### Comparison with direct regressions

At medium to very high N concentrations, the estimation from GAM for the full set of streams was different from the dose-response estimation by the generalized propensity score (Fig. 4). However, among the three sub-ecoregions, the difference between the direct regression and the generalized propensity score (Fig. 5) is smaller than the difference shown in Fig. 4. In the Northwestern Forested Mountains stratum, GAM had fit a subsidy-stress relationship that was only slightly outside the 95% confidence zone of the generalized propensity score estimation when within the middle ranges of N concentration (Fig. 5a). For the North American Deserts stratum, the GAM fit completely within the 95% confidence zone of the generalized propensity score (Fig. 5c). For the Great Plains stratum, the fitted lines from both the simple and multiple linear regressions closely followed the mean estimation of the generalized propensity score (Fig. 5b).

## Discussion

On average, benthic invertebrate richness in streams within the 12 western states of the USA exhibits a subsidy-stress relationship with total nitrogen. This result is consistent with the conclusion that a high level of nutrient concentrations negatively affects invertebrate richness in streams, a conclusion also found in manipulative experiments and observational studies (Tilman 1987; Dodson et al. 2000; Haddad et al. 2000; Wang et al. 2007). Yuan (2010) studied the same data from the EPA by stratifying the data into different groups based on predicted log(TN) concentrations from its covariates instead of the propensity score. The six groups essentially represent different N concentration ranges. Yuan’s (2010) observed increases in TN were associated with small increases in IR in the first three groups (low nutrient levels) and a significantly negative correlation between log TN and IR in the last three groups (high nutrient levels), suggesting that there is a subsidy-stress relationship. The average subsidy-stress relationship found over a large region also explains why conflicting results (e.g., no correlation, positive and negative association) have been found in small areas. When streams in a small area have limited nutrients, increases in nutrients can lead to increase in periphyton biomass, which can support a greater diversity of invertebrates (Chetelat et al. 1999); therefore, only positive correlations could be observed. On the other hand, when nutrients are excessive in a stream, the diminished water quality and the depleted oxygen caused by decomposition of periphyton biomass will likely reduce IR (Correll 1998), leading to a negative association between nutrients and IR.

Although the same subsidy-stress relationship was found in the three ecoregions we studied, the variation among regions is also obvious. At a similar nutrient level, streams in different ecoregions have different expected IR (Fig. 5), as nutrient enrichment is only one of the many factors that may influence the diversity of the stream macroinvertebrate community (Wang et al. 2007; Yuan 2010). The generalized propensity score produces an average dose-response function that is controlled by the average condition represented in the data. The three strata in our study have different covariate mean values (Table 2), representing differences in natural conditions and anthropogenic activities; the differences in these conditions and activities contribute to the variability in both the observed nutrient concentrations and the TN-IR relationship. For example, both local- and catchment-scale disturbances can reduce stream macroinvertebrate taxon richness (Ligeiro et al. 2013). The Great Plains ecoregion has the highest percentage of agricultural and urban areas and riparian disturbance. This stratum thus has the lowest IR, even for streams at comparable TN levels with streams in other strata. The characteristics of flow regimes affected by precipitation events are widely recognized as important variables in determining the diversity and community composition in streams (Resh et al. 1988; Poff et al. 1997). Different precipitation patterns in the three ecoregions can produce totally different flow regimes (David 2006) that may change the nutrient-IR relationship (Palardy and Witman 2010). Higher temperatures in the North American Deserts can also increase stream temperature, changing sediments, and dissolved oxygen (Darren et al. 2013) which may increase the excessive nutrient stress to IR (USEPA 1996). Similar effects of other covariates on IR and nutrients may also occur.

The detrimental effects of excess nutrients require controls on nutrient loadings to streams based on nutrient effects (Dodds and Welch 2000). Our study provides practical guidance on how to find a scientifically defensible threshold value of TN (e.g., the breaking point log (TN) in Fig. 3b) that will result in no detectable harmful effect on IR. If confounding effects were not approximately controlled, the resulting biased estimate of the causal effect would lead to a biased threshold value. It is therefore important to have reliable estimates of causal effects. The same approach and principle can be applied to other pollutants and ecological indicators. However, as varying natural and anthropogenic conditions exist in different regions, nutrient effects are stream- or region-specific (Fig. 5). As a result, nutrient thresholds for setting environmental standards need to be region/area-specific to increase their value for application. For example, a nationwide nutrient threshold might be higher for some states/provinces but lower for other states/provinces, and the same problem can be extended to different counties within a state/province. However, in practice, the sample size may be limited when the area of targeted regions becomes small, which may increase the uncertainty associated with dose-response estimation. The value of larger-scale studies like the current study can be realized under a Bayesian framework, where an informative prior distribution of the TN effect can be derived (Gelman and Hill 2007). This use of the Bayesian approach is consistent with the interpretation of a Bayesian prior distribution representing the among-site variability (Qian et al. 2015). Local agencies can establish a process whereby they gradually update the dose-response function when new region-specific data are available. This Bayesian updating approach will gradually move from the larger scale average dose-response relationship towards a locally region-specific dose-response relationship.

An important assumption of the generalized propensity score approach is the “weak unconfoundedness” assumption, which states that all important confounding factors are included in the treatment assignment model (i.e., Eqs. (3) and (4)). This assumption suggests that we should collect as many covariates as possible when designing an observational study to guard against missing any important confounding factors. We have identified and included all important confounders available from the wadeable stream assessment dataset, which was created by the EPA (Table 1). However, we may have missed some potentially important confounding factors that were not observed, such as site history and variability of flow velocity. Nevertheless, we have controlled some of the potential covariates and strengthened the estimation of the nutrient’s causal effect on invertebrate richness. Many unobserved confounding factors are likely to be correlated with these observed covariates and are thus partially controlled for as well.

When we started this study, we expected that the dose-response relationship estimated by GPS would be different from that estimated by linear regression and GAM. The difference is indeed obvious when comparing the two estimated dose-relationships using the entire dataset (Fig. 4, top panel). However, the differences were not obvious when we repeated the same modeling approaches in the three ecoregion-based strata. When using the entire dataset, confounding factors are highly imbalanced. This imbalance is shown in the large (absolute value) standardized mean differences of all confounding factors (Table 3). Such imbalances are, however, not as pronounced in the data within the three ecoregion-based strata (Tables 4, 5, and 6). As a result, ecoregion-based dose-response relationships are relatively similar, with or without GPS adjustment. This result highlights the regional differences in the dose-response relationship, which suggests the value of region-specific nutrient criteria.

## Conclusions

The regional average of nutrient causal effects on stream invertebrate richness was estimated using observational data, with confounding effects controlled for using the generalized propensity score. The aggregated confounding effects were removed by integration through the single propensity score. We found a subsidy-stress relationship between nutrients and invertebrate taxon richness across streams both in the Western United States and its sub-regions. This same general pattern varies among ecoregions due to the varying natural conditions and anthropogenic activities. The variation demonstrated that invertebrates respond to the same nutrient levels differently across different conditions.
